# Additional data on the investigation of the reaction mechanisms for the production of silica hyperbranched polyethylene imine silver nanoparticle composites

**DOI:** 10.1016/j.dib.2022.108374

**Published:** 2022-06-15

**Authors:** Michael Arkas, Marilina Douloudi, Eleni Nikoli, Georgia Karountzou, Ioanna Kitsou, Eleni Kavetsou, Dimitrios Korres, Stamatina Vouyiouka, Athena Tsetsekou, Konstantinos Giannakopoulos, Michaela Papageorgiou

**Affiliations:** a Institute of Nanoscience Nanotechnology NCSR “Demokritos”, Patriarchou Gregoriou Str., Aghia Paraskevi, Athens 15310, Greece; b School of Mining Engineering and Metallurgy, National Technical University of Athens, Iroon Polytechneiou 9, Athens 15780, Greece; c School of Chemical Engineering, National Technical University of Athens, Iroon Polytechneiou 9, Athens, Athens 15780, Greece

**Keywords:** Dendritic polymers, Dendrimers, Hyperbranched polymers, Catalysis, Composite materials, Biomimetic chemistry, Hybrid materials, Metal nanoparticles

## Abstract

Silica-organic matrix-silver, nano-catalysts, were synthesized employing four different hyperbranched poly(ethylene imines) (MW 2000 to 750,000) to reduce Ag^+^ to metal nanoparticles and the formation of formation SiO_2_ shells. The latter is performed at pH 7,5 employing three different pH regulating agents Hepes, Trizma, and Phosphate Salts. Characterization of the resulting materials with spectroscopy (FTIR), thermogravimetry (TG), scanning electron microscopy (SEM), and ζ-potential is reported. Kinetic studies of standard reactions, 4-nitrophenol and 4-nitroaniline reduction to 4-aminophenol and p-phenylenediamine, respectively by UV-Visible spectroscopy are also included. This data in brief article is related to the “Investigation of two Bioinspired Reaction Mechanisms for the Optimization of Eco Composites-Nano Catalysts Generated from Hyperbranched Polymer Matrices” manuscript submitted to reactive & functional polymers.

## Specifications Table

**Table:** 

Subject	Chemistry Chemical Engineering: Catalysis
Specific subject area	Composite Metal Nanocatalysts
Type of data	Tables Figures
How data were acquired	UV-Visible spectroscopy - Cary 100 UV–visible spectrophotometer, Thermogravimetry - Mettler Toledo TGA/DSC 1 System, Scanning Electron Microscopy (SEM) - FEI Inspect microscope
Data format	Raw Data csv and pdf format Analyzed Data Figures and Tables
Description of data collection	For the thermogravimetric analysis, the samples were heated from 25 to 700 °C under nitrogen flow (10 ml/min), heating rate of 10 °C/min, and then remained at this temperature for 3 h. For the UV-Visible spectroscopy: To avoid the scattering due to the presence of the dispersions of the catalysts and H_2_ bubbles, reaction solutions without the nitro-compounds were employed as reference instead of water.
Data source location	Institution: Institute of Nanoscience Nanotechnology NCSR “Demokritos” City/Town/Region: Aghia Paraskevi/Athens/Attica Country: Greece Latitude and longitude and GPS coordinates, for collected samples/data: 37°59′47.5"N 23°49′01.3″E 37.996538, 23.817030 Institution: School of Mining Engineering and Metallurgy, National Technical University of Athens City/Town/Region: Zografou/Athens/Attica Latitude and longitude and GPS coordinates, for collected samples/data: 37°58′32.3″N 23°46′58.3″E 37.975625, 23.782848 Institution: School of Chemical Engineering, National Technical University of Athens City/Town/Region: Zografou/Athens/Attica Latitude and longitude and GPS coordinates, for collected samples/data: 37°58′35.5″N 23°47′06.3″E 37.976532, 23.785080
Data accessibility	Thermogravimetry Data: Repository name: Mendeley Data Data identification number: DOI: 10.17632/22symh9gs4.2 Direct URL to data: https://data.mendeley.com/datasets/22symh9gs4/2
Related research article	Arkas et al. Investigation of two Bioinspired Reaction Mechanisms for the Optimization of Eco Composites-Nano Catalysts Generated from Hyperbranched Polymer Matrices. https://doi.org/10.1016/j.reactfunctpolym.2022.105238

**Value of the Data**

• SEM micrographs provide correlation on composites shape/size in relation with the preparation method.
• Every researcher in fields relative to composite materials could take advantage of the reported data.
• These data may be used for the development of similar composite catalysts with other metals and shells.
• Thermogravimetry Data provide important information on the material's composition.

## 1 Data Description

Table 1 explains the nomenclature of the samples: the numbers represent the MW of hyperbranched poly(ethylene imine) P, T, H represents the pH regulating agent, Ag the presence of silver, and C the optional calcination step. Fig. 1, Fig. 2, Fig, 3, Fig. 4, Fig. 5, Fig. 6, Fig. 7, Fig. 8, Fig. 9, Fig. 10, Fig. 11, Fig. 12, Fig. 13, Fig. 14, Fig. 15 are representative SEM micrographs of PEI-silica composite nanospheres and PEI-silica-Ag nanocatalysts. Fig. 16, Fig. 17, Fig. 18, Fig. 19, Fig. 20, Fig. 21, Fig. 22, Fig. 23, Fig. 24, Fig. 25, Fig. 26 are TGA results and the respective 1st derivatives of PEI-silica nanospheres and PEI-silica-Ag nanocatalysts. Raw data are publicly available on the Mendeley Data repository https://data.mendeley.com/datasets/22symh9gs4/1 [5]. Fig. 27 is a diagram of the w/w% compositions of the PEI-silica nanospheres and the PEI-silica-Ag nanocatalysts (raw data values included in the diagram). Fig. 28 contains the nitrophenol and nitroaniline reduction rate coefficients and Table 2 contains the raw data.

**Table 1:** Sample classification according to the MW of PEI, the buffering agent, the presence of Ag, and the employment of an optional calcination step at 700 °C 'modified from [1].

PEI-Silica Nanospheres Mw	Phosphates	Trizma	Hepes
2000	2000-P	2000-T	2000-H
5000	5000-P	5000-T	5000-H
25,000	25,000-P	25,000-T	25,000-H
750,000	750,000-P	750,000-T	750,000-H

PEI-Silica-Ag Nanocatalysts Mw			
2000	Ag-2000-P	Ag-2000-T	A-2000-H
5000	Ag-5000-P	Ag-5000-T	Ag-5000-H
25,000	Ag-25,000-P	Ag-25,000-T	Ag-25,000-H
750,000	Ag-750,000-P	Ag-750,000-T	Ag-750,000-H

Calcinated-PEI-Silica-Ag Nanocatalysts Mw			
2000	Ag-2000-P-C	Ag-2000-T-C	A-2000-H-C
5000	Ag-5000-P-C	Ag-5000-T-C	Ag-5000-H-C
25,000	Ag-25,000-P-C	Ag-25,000-T-C	Ag-25,000-H-C
750,000	Ag-750,000-P-C	Ag-750,000-T-C	Ag-750,000-H-C

**Fig. 1:**
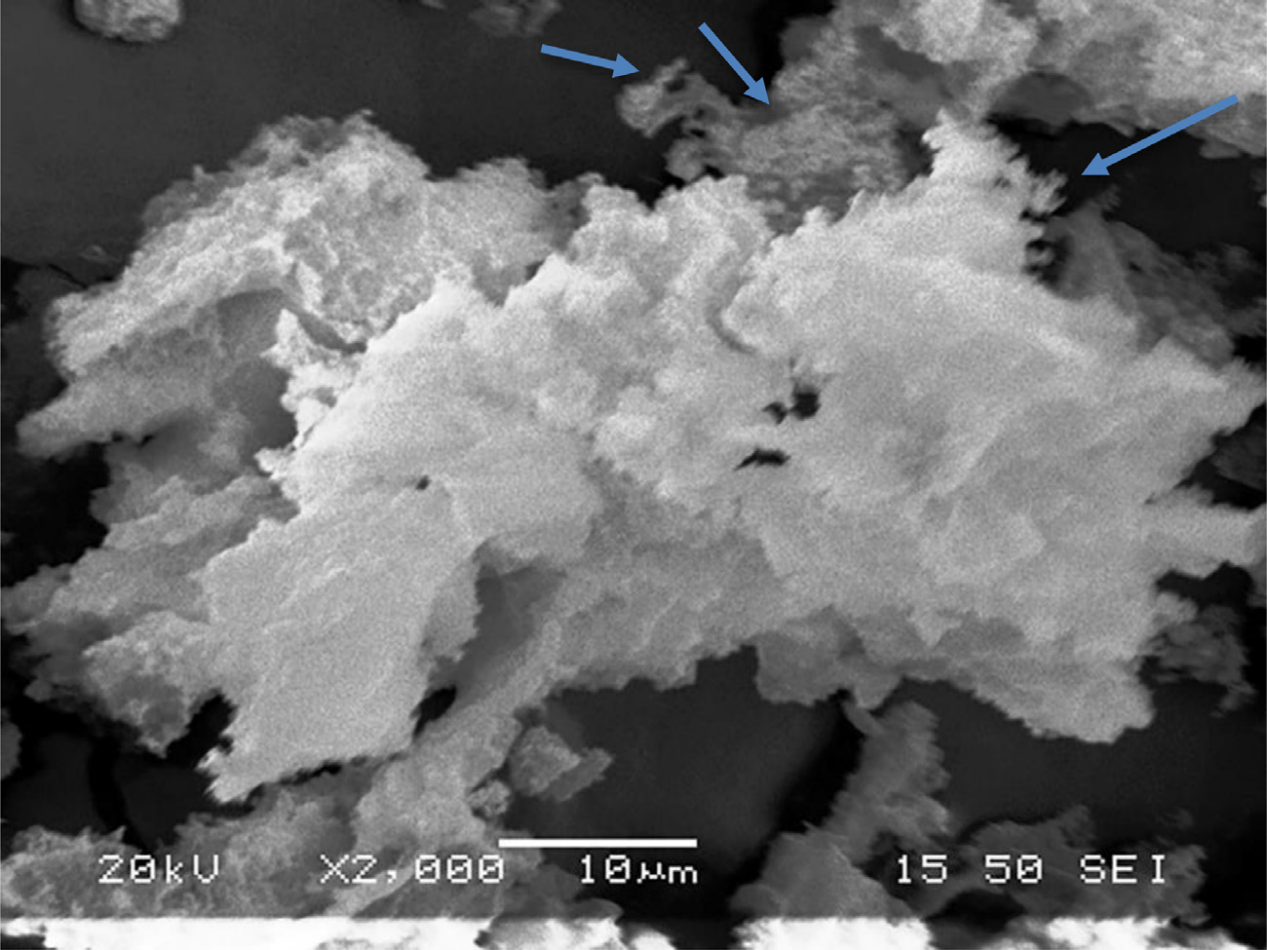
SEM micrograph of 2000-H. Published with permission from [1].

**Fig. 2:**
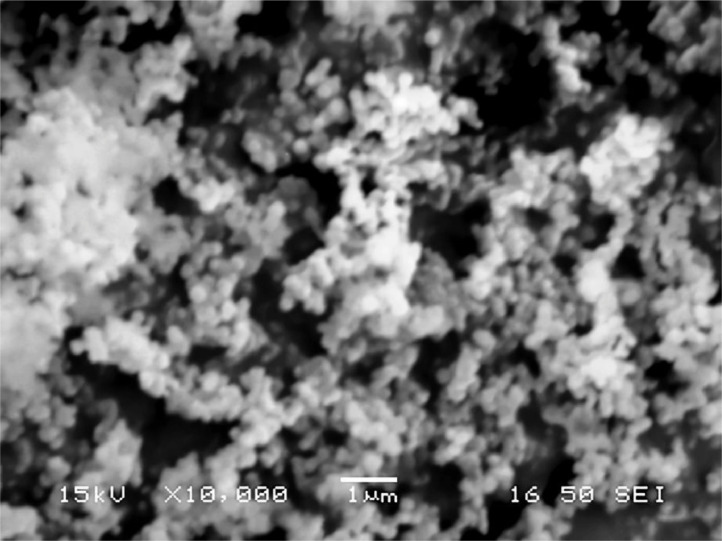
SEM micrograph of 2000-P. Published with permission from [1].

**Fig, 3:**
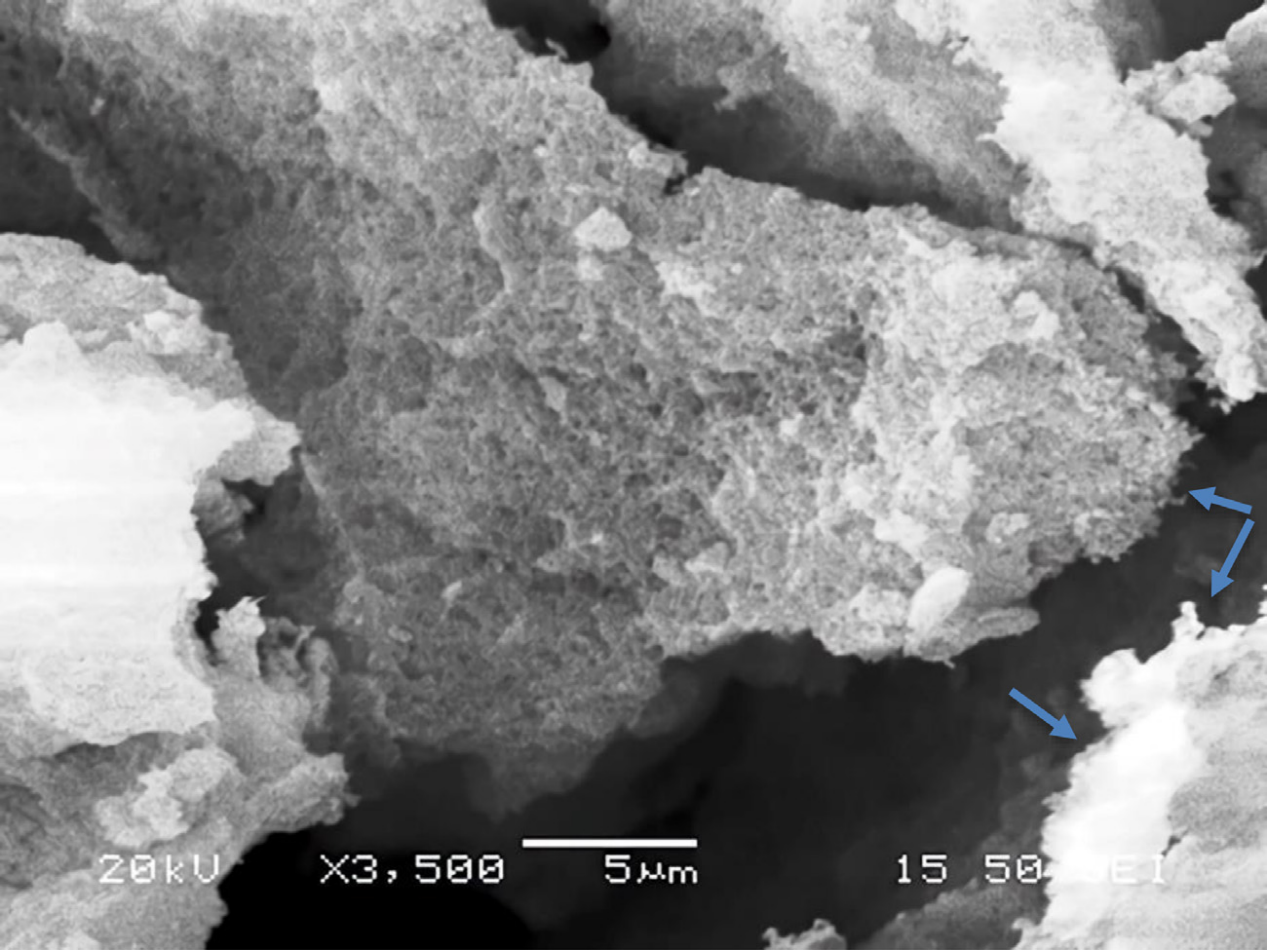
SEM micrograph of 2000-T. Published with permission from [1].

**Fig. 4:**
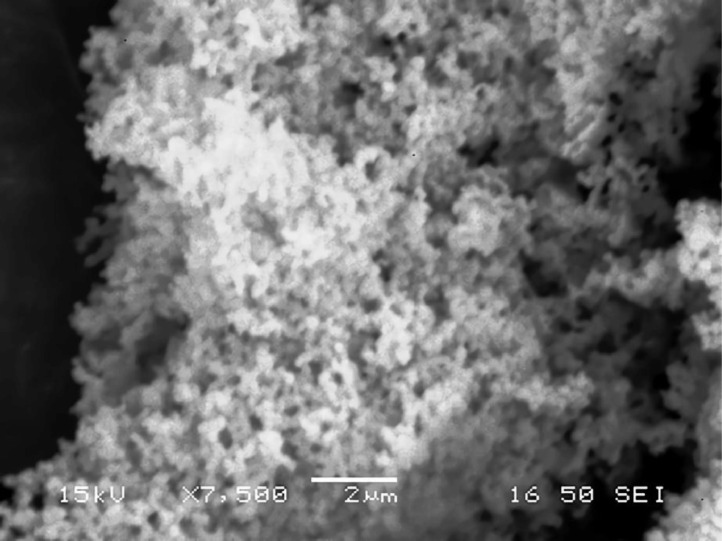
SEM micrograph of 5000-P. Published with permission from [1].

**Fig. 5:**
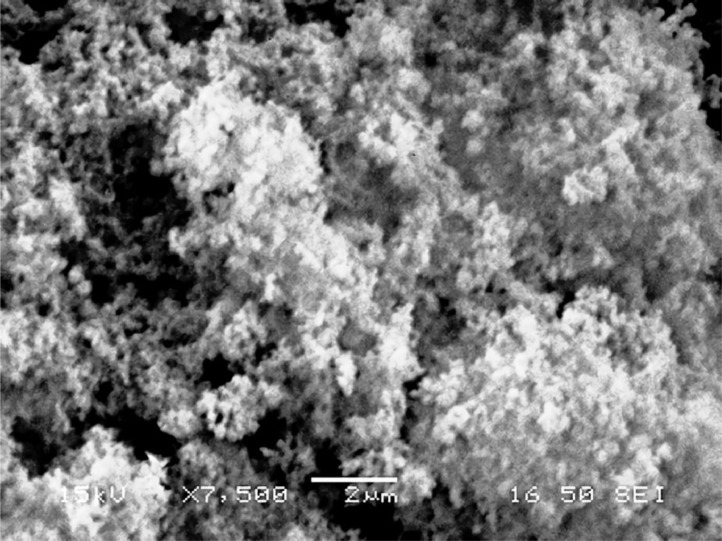
SEM micrograph of 25,000-P. Published with permission from [1].

**Fig. 6:**
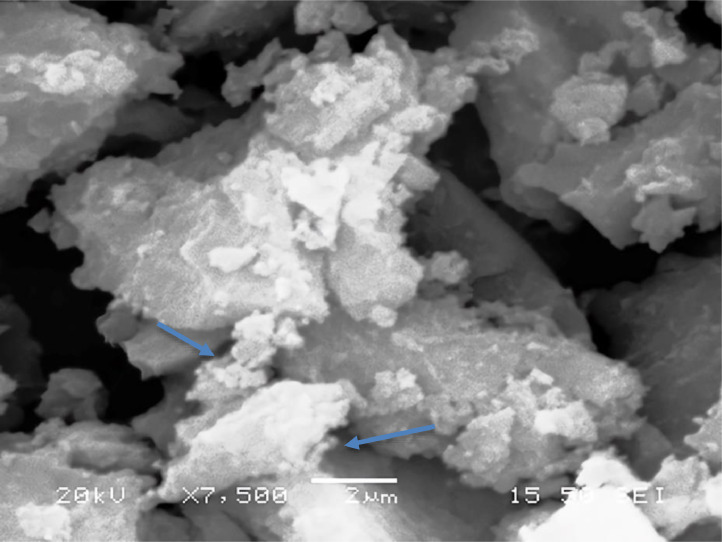
SEM micrograph of 25,000-T.

**Fig. 7:**
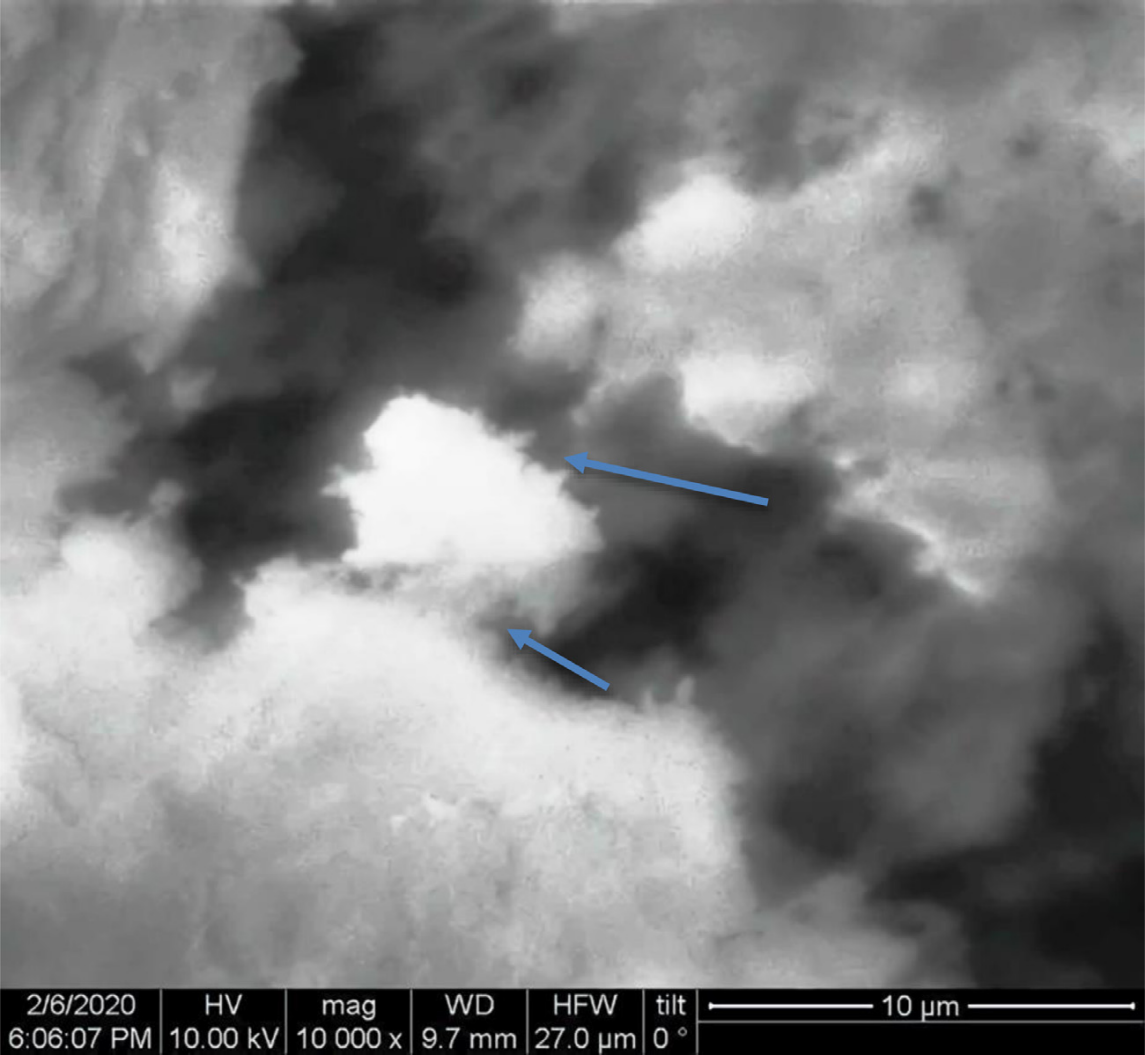
SEM micrograph of 750,000-H. Published with permission from [1].

**Fig. 8:**
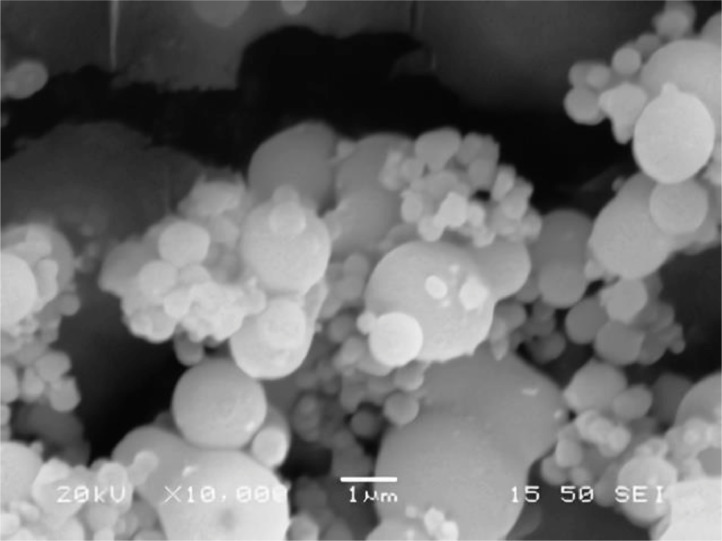
SEM micrograph of 750,000-P. Published with permission from [1].

**Fig. 9:**
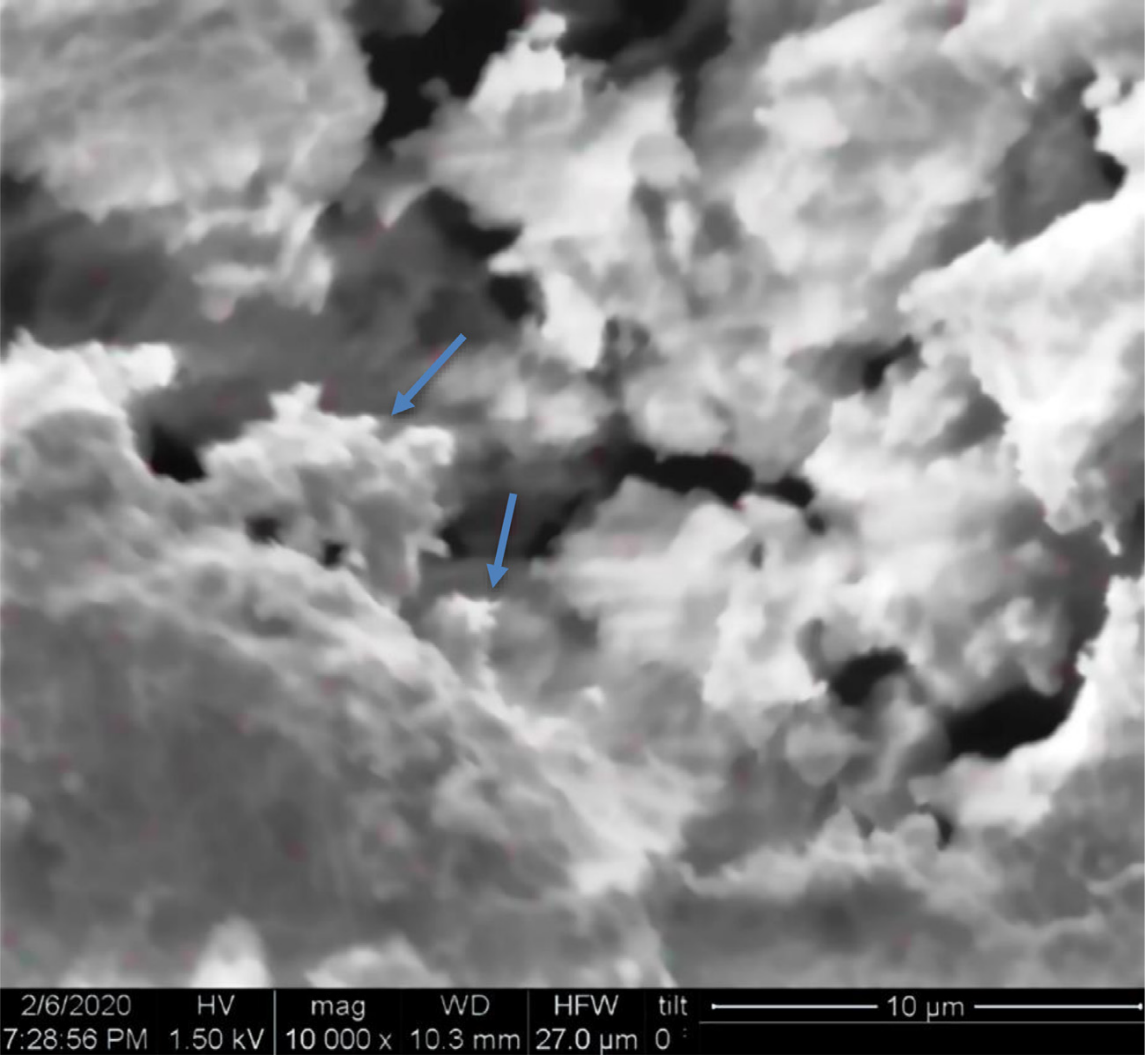
SEM micrograph of 750,000-T. Published with permission from [1].

**Fig. 10:**
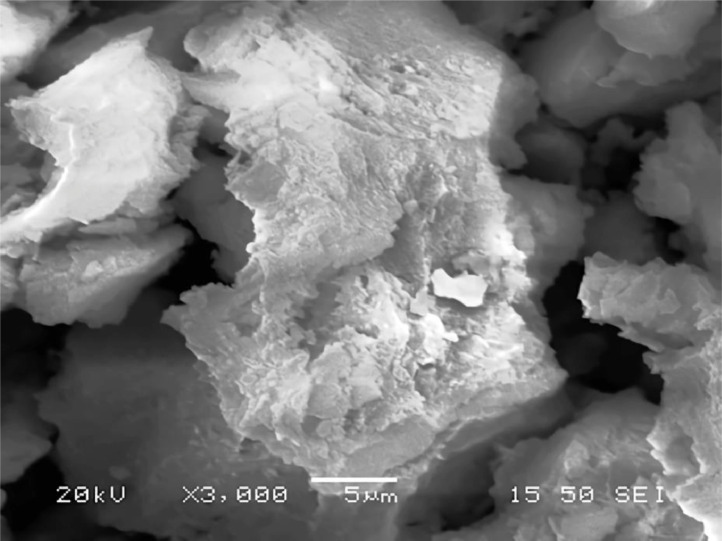
SEM micrograph of Ag-2000-H.

**Fig. 11:**
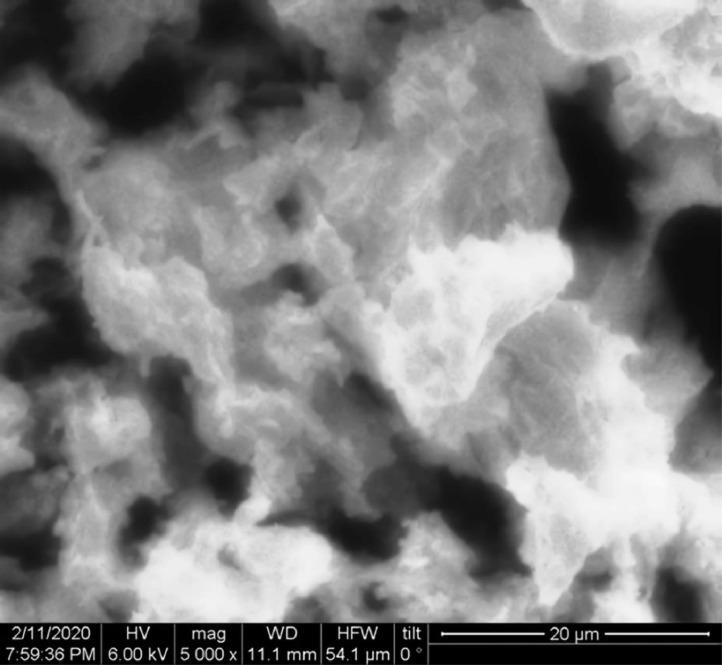
SEM micrograph of Ag-2000-P.

**Fig. 12:**
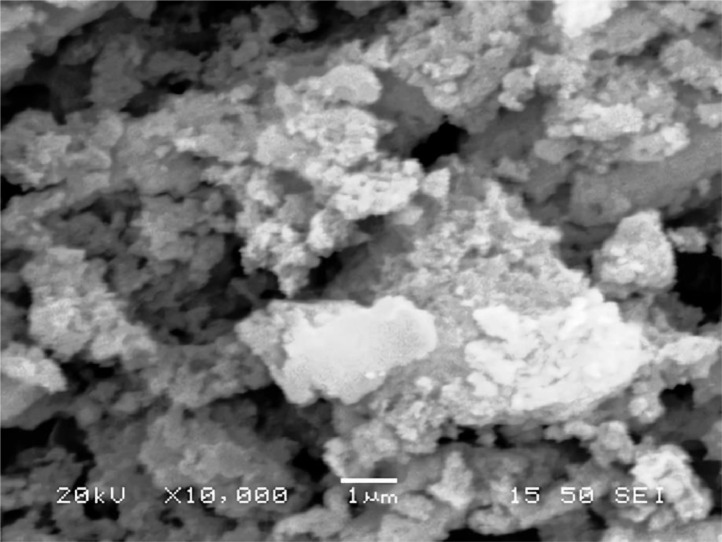
SEM micrograph of Ag-5000-H. Published with permission from [1].

**Fig. 13:**
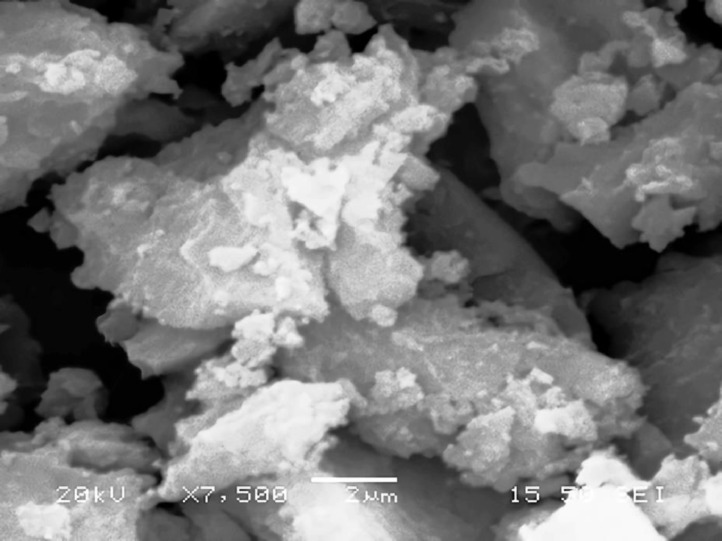
SEM micrograph of Ag-25,000-H.

**Fig. 14:**
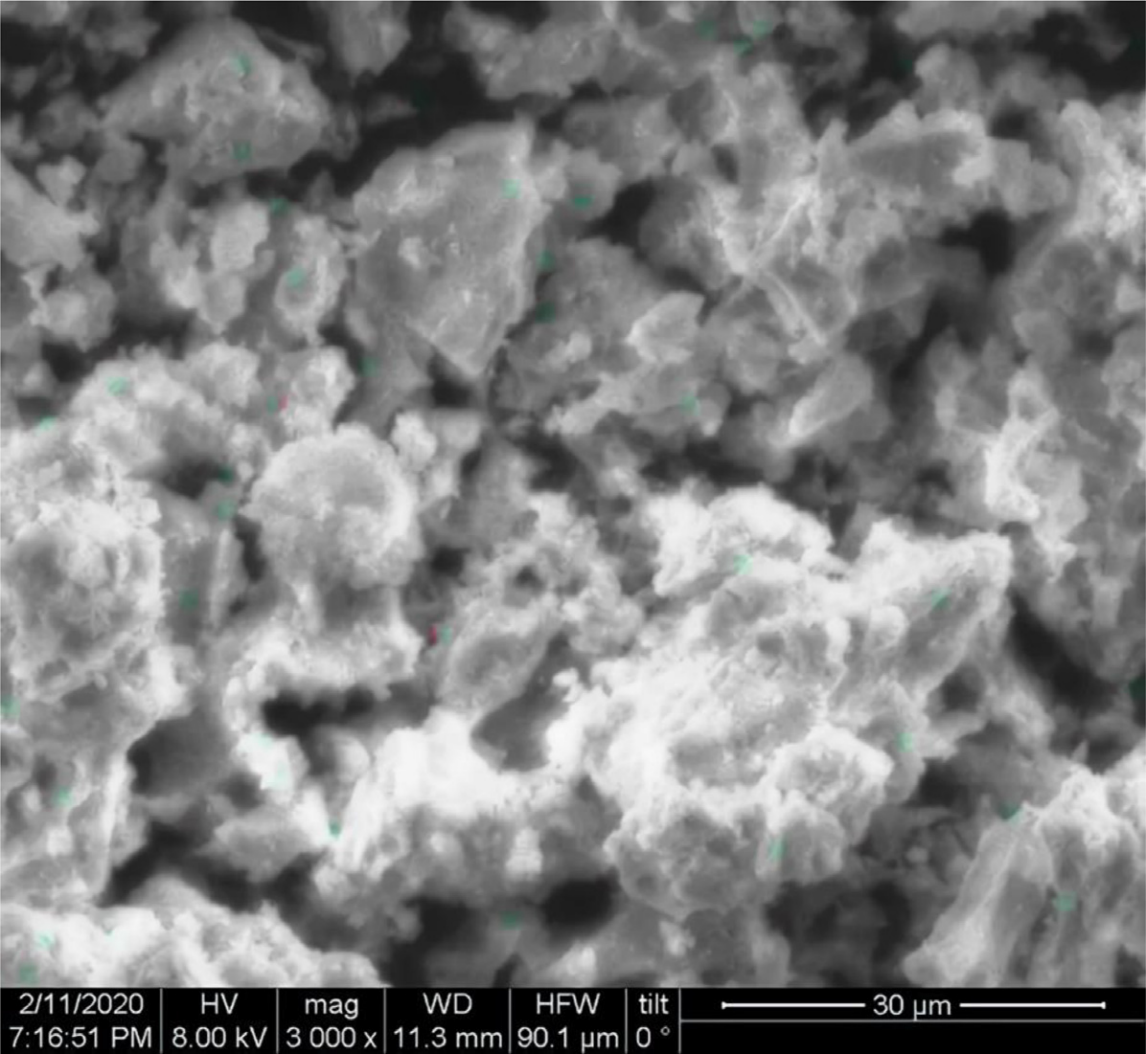
SEM micrograph of Ag-25,000-T.

**Fig. 15:**
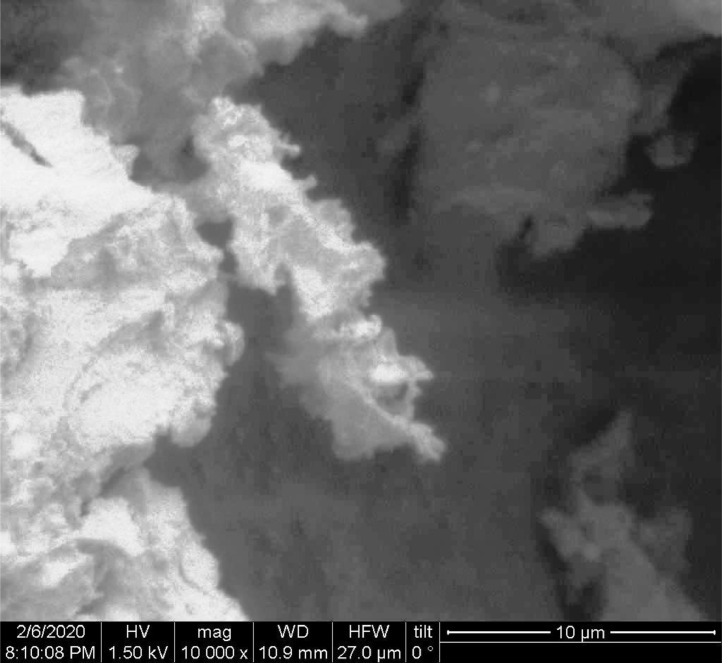
SEM micrograph of Ag-750,000-T. Published with permission from [1].

**Fig. 16:**
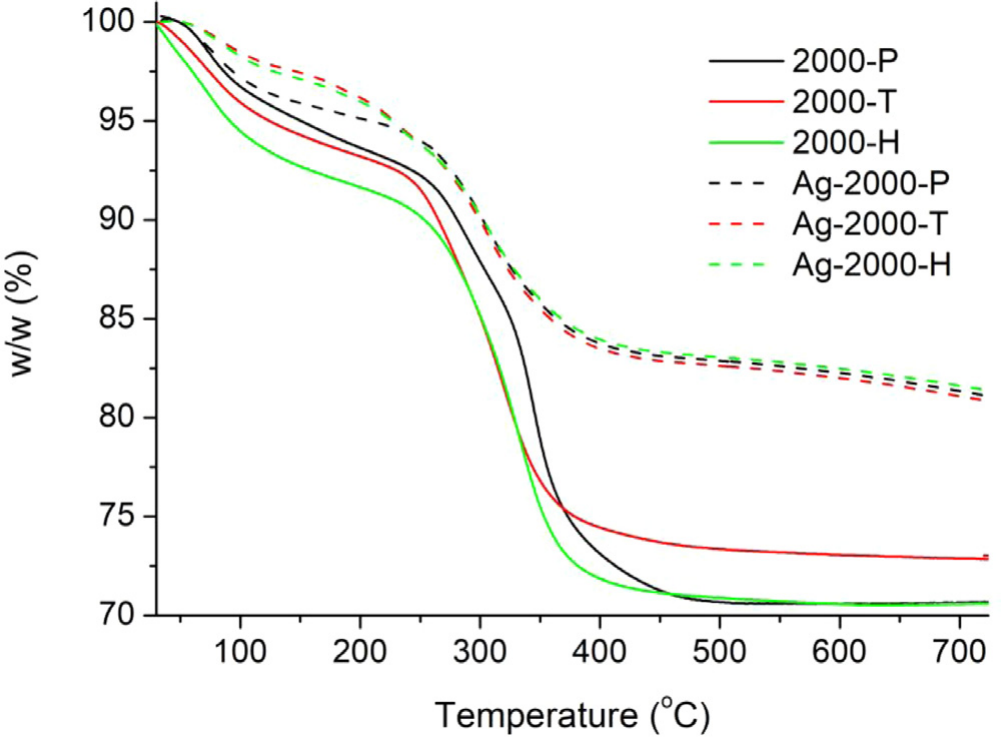
Mass percentage of silica-PEI 2000 composites as a function of temperature.

**Fig. 17:**
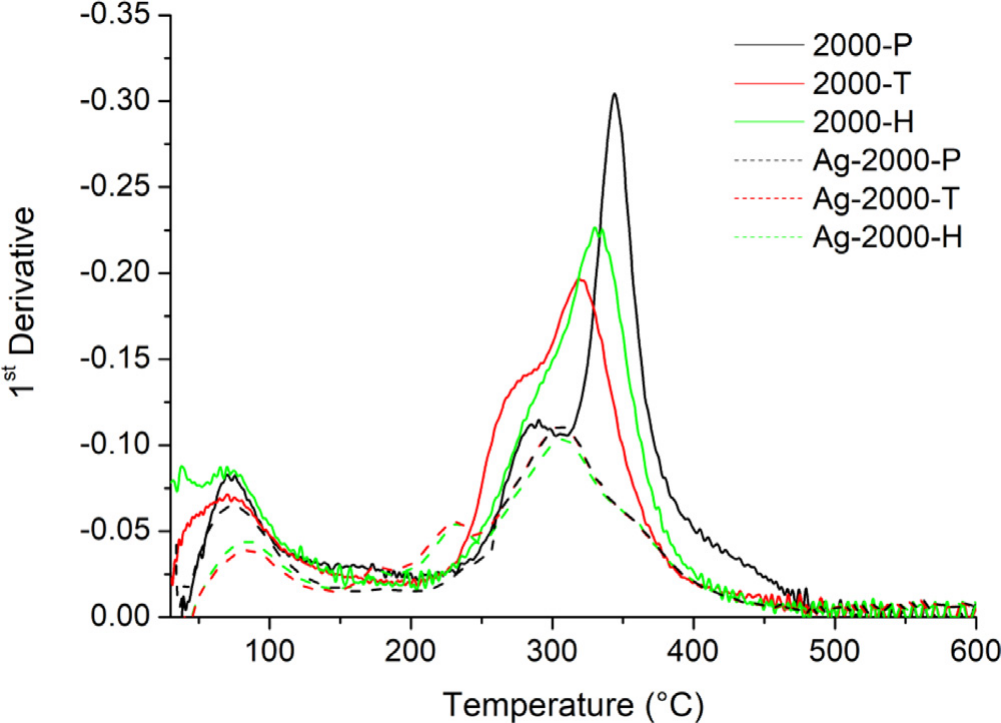
First derivative of the mass percentage of silica-PEI 2000 composites as a function of temperature.

**Fig. 18:**
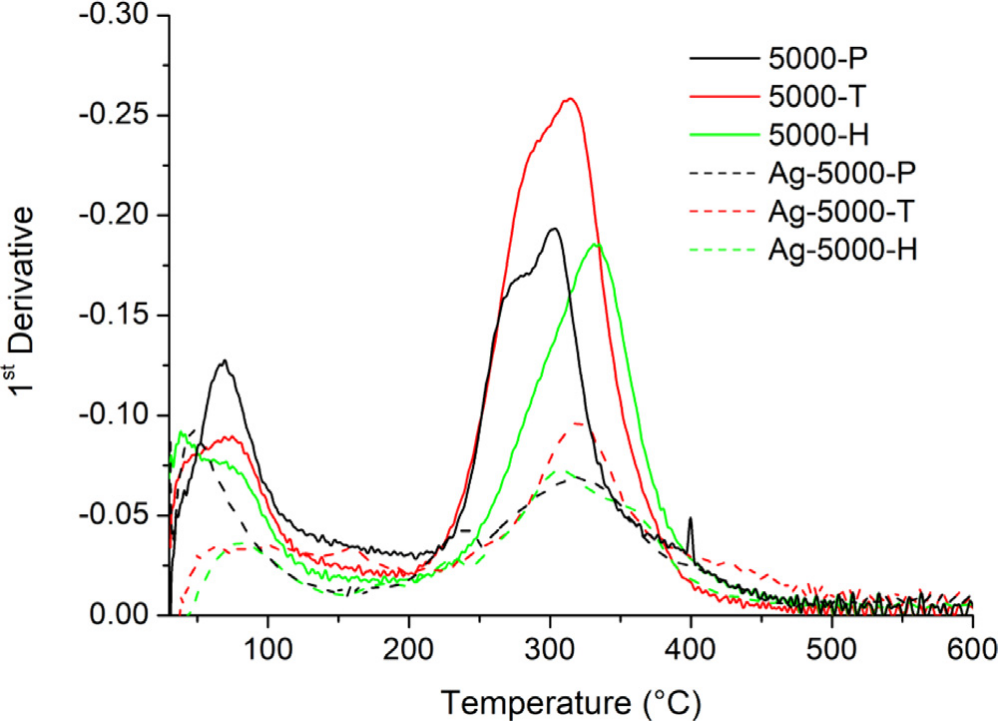
First derivative of the mass percentage of silica-PEI 5000 composites as a function of temperature.

**Fig. 19:**
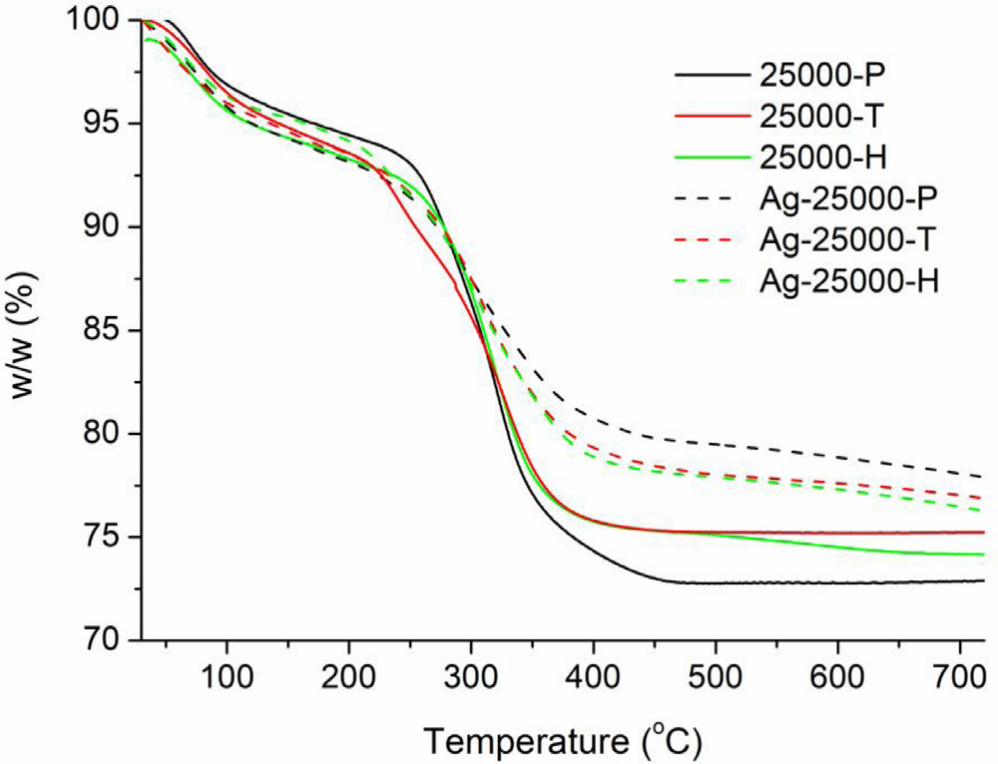
Mass percentage of silica-PEI 25,000 composites as a function of temperature.

**Fig. 20:**
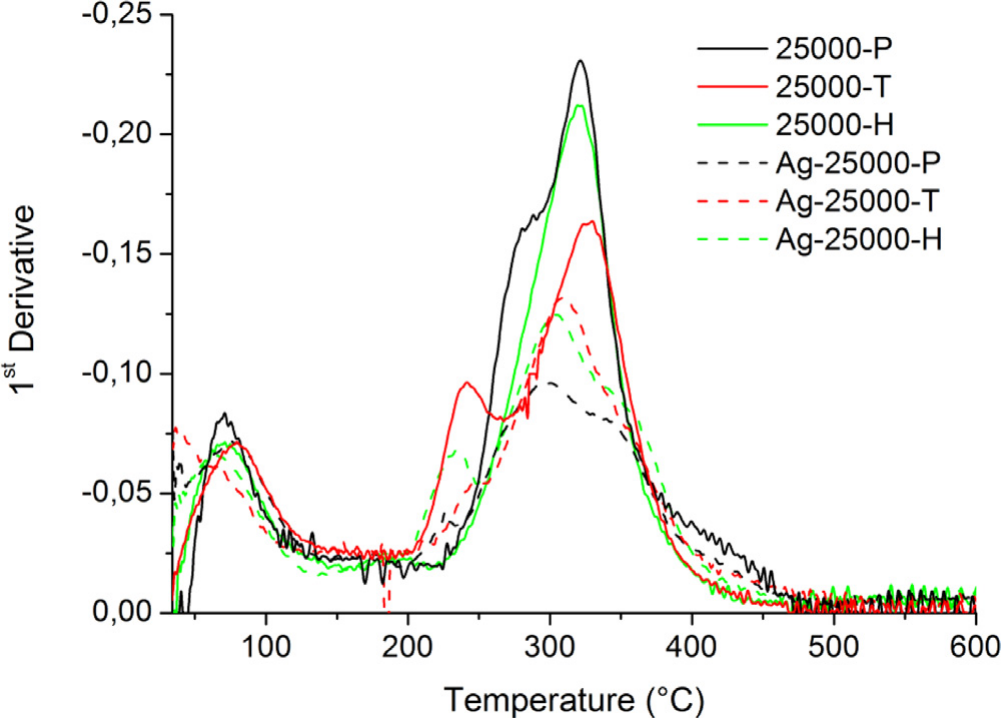
First derivative of the mass percentage of silica-PEI 25,000 composites as a function of temperature.

**Fig. 21:**
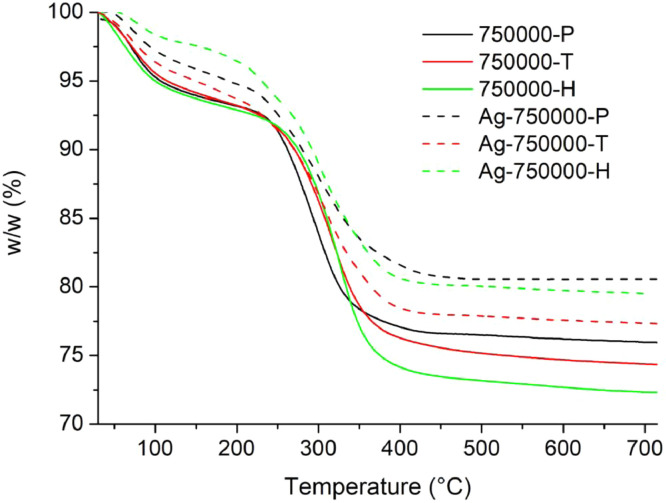
Mass percentage of silica-PEI 750,000 composites as a function of temperature.

**Fig. 22:**
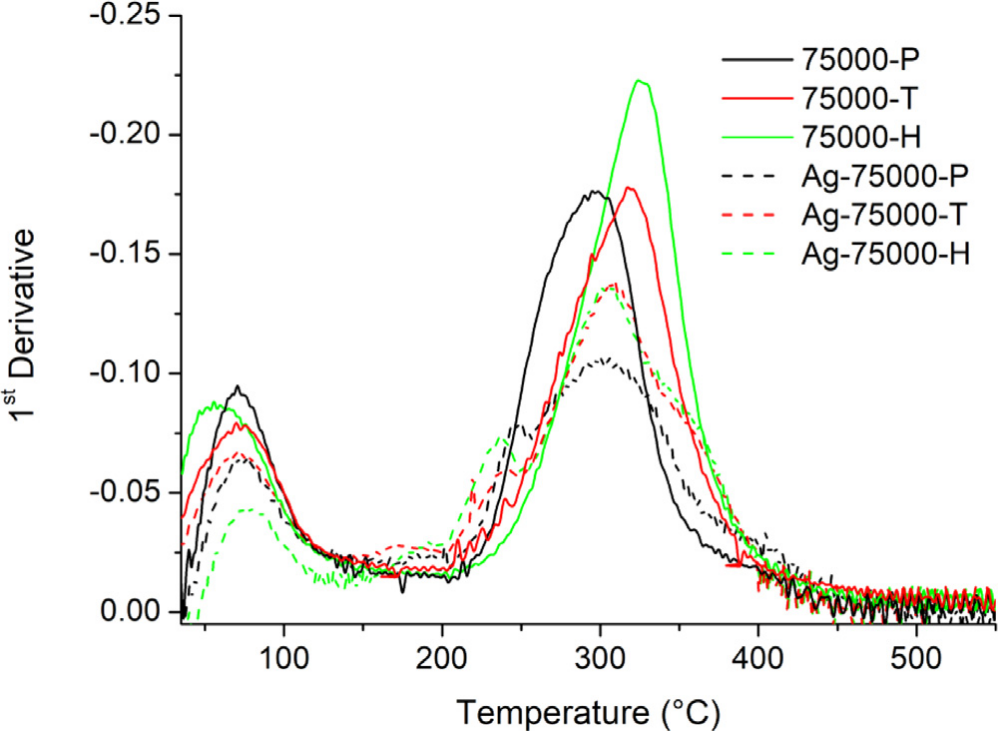
First derivative of the mass percentage of silica-PEI 750,000 composites as a function of temperature.

**Fig. 23:**
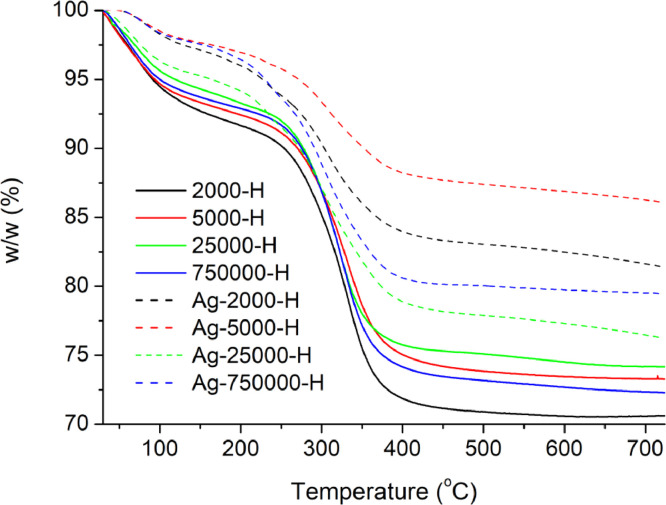
Mass percentage of silica-PEI composites prepared by Hepes as a function of temperature.

**Fig. 24:**
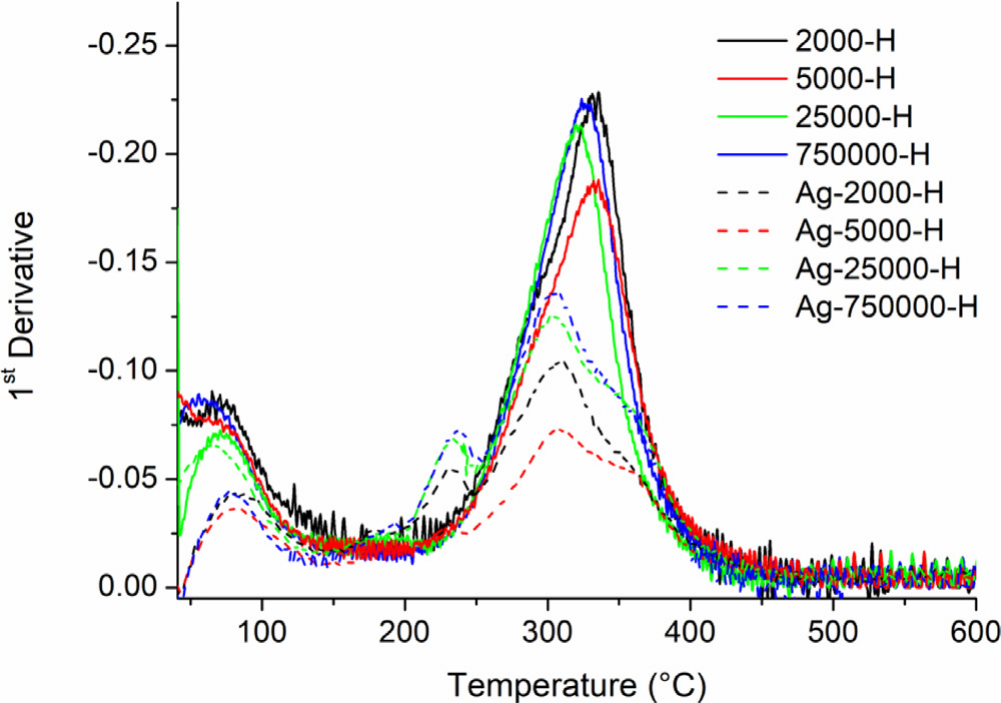
First derivative of the mass percentage of silica-PEI composites prepared by Hepes as a function of temperature.

**Fig. 25:**
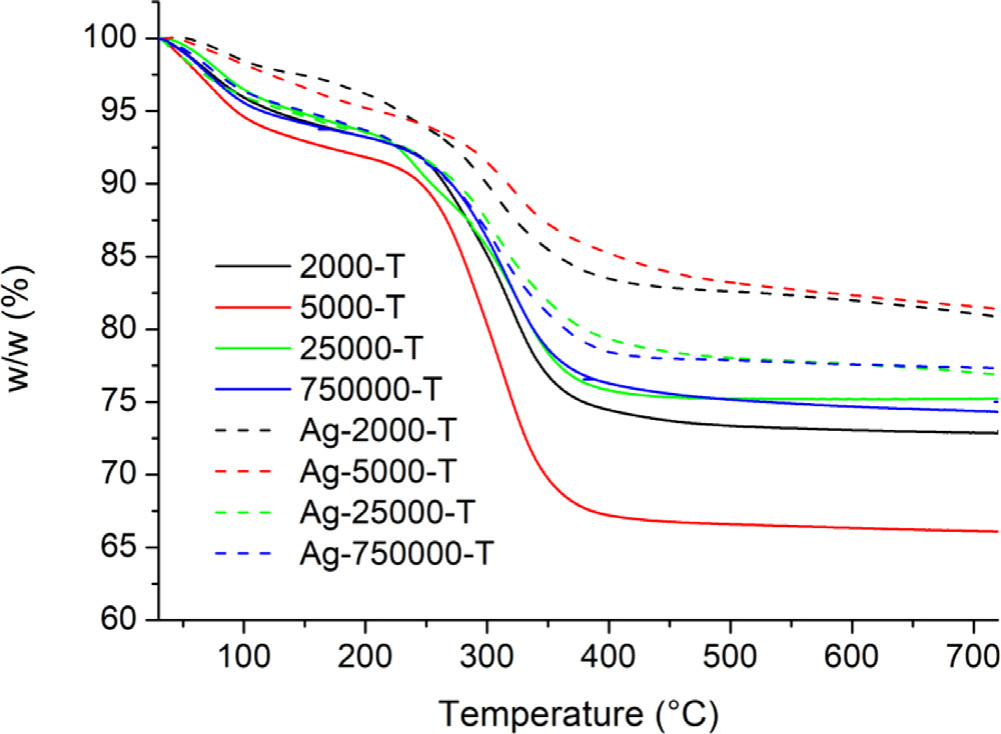
Mass percentage of silica-PEI composites prepared by Trizma as a function of temperature.

**Fig. 26:**
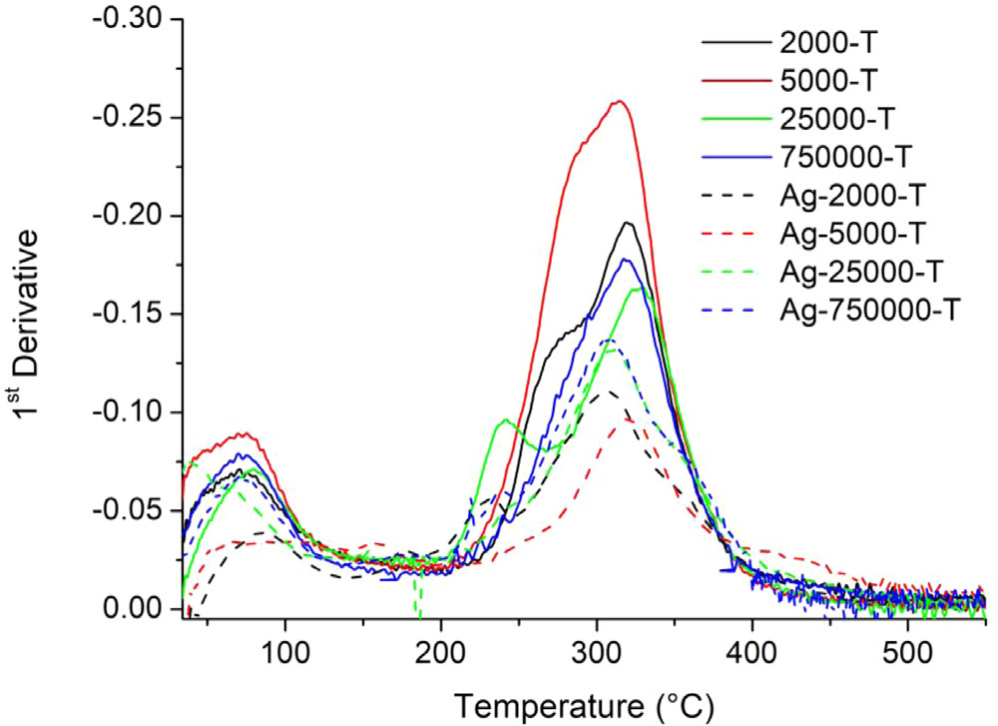
First derivative of the mass percentage of silica-PEI composites prepared by Trizma as a function of temperature.

**Fig. 27:**
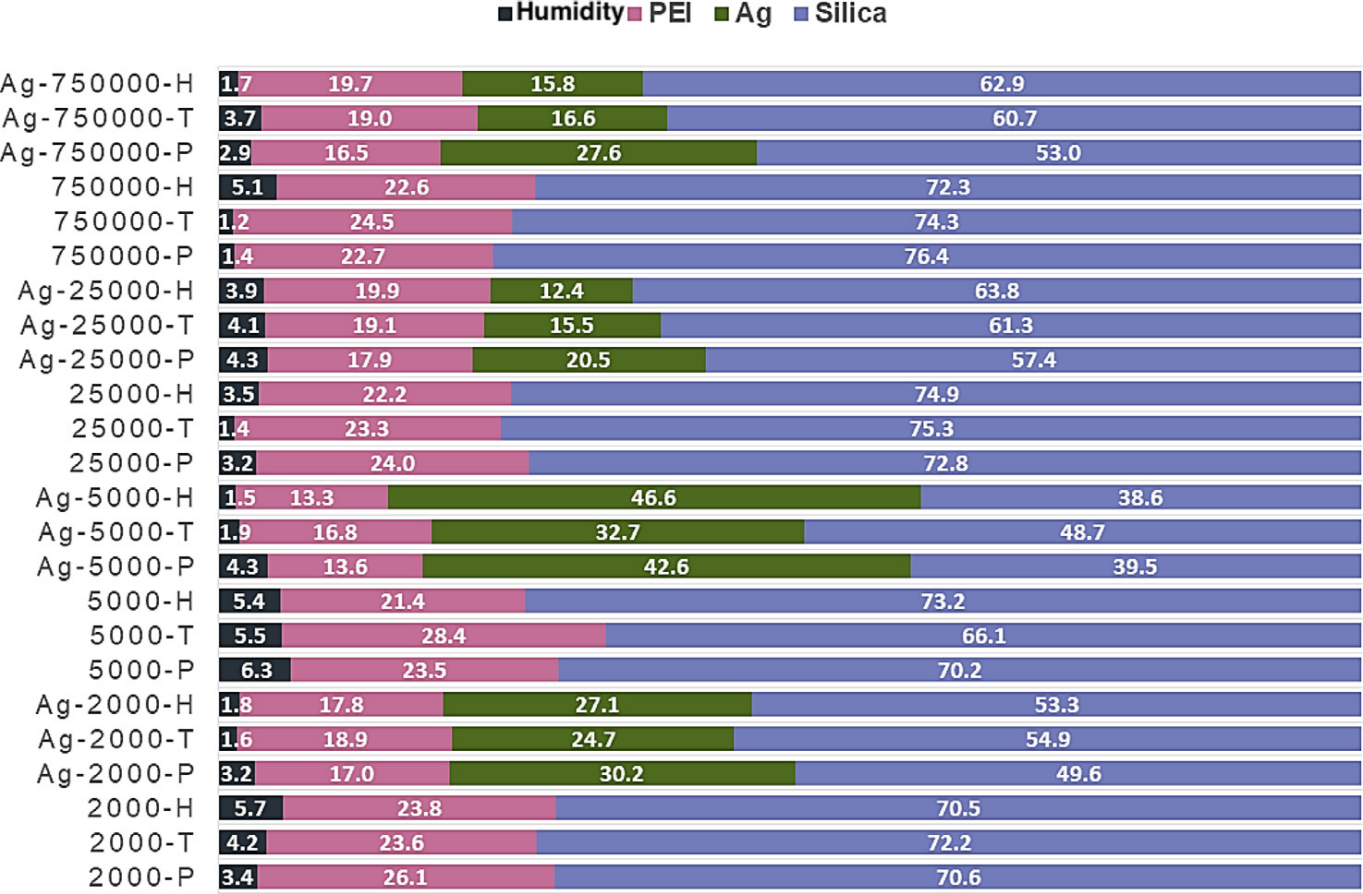
Composition (w/w%) of PEI-silica and Ag-PEI-silica nanocomposites.

**Fig. 28:**
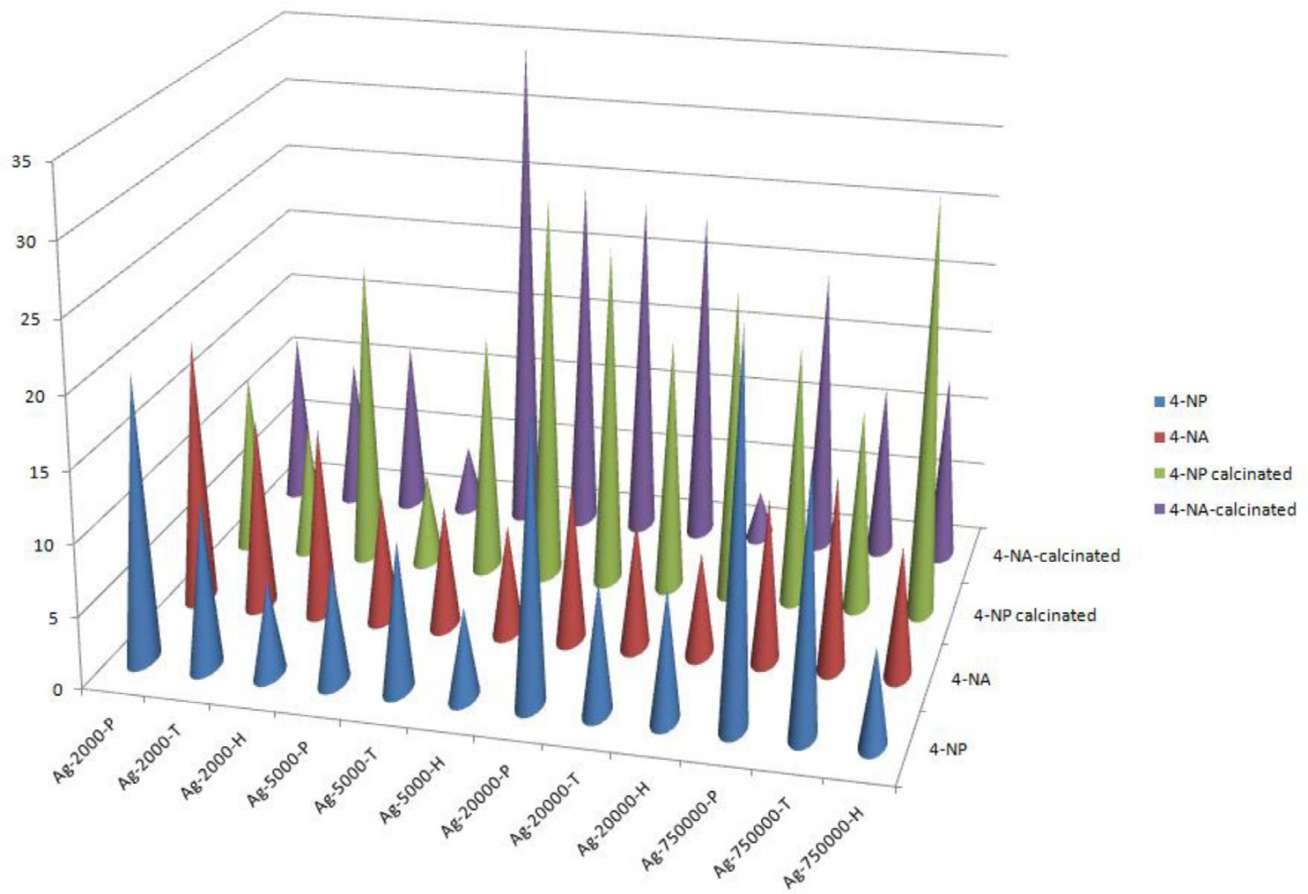
Reduction rate coefficients (x -10^3^) for all the composite catalysts.

**Table 2:** Reaction rate coefficients and R-squared values for the composite catalysts.

	4NP		4NP	
Sample	k (s^−1^)	R^2^	k (s^−1^)	R^2^
Ag-2000-H	0,0066	0,98603	0,0131	0,95033
Ag-2000-T	0,0117	0,97281	0,0133	0,92117
Ag-2000-P	0,0199	0,94508	0,0186	0,93312
Ag-5000-H	0,0062	0,97534	0,0076	0,9622
Ag-5000-T	0,0103	0,99668	0,0085	0,91881
Ag-5000-P	0,0082	0,94446	0,0089	0,93267
Ag-25,000-H	0,009	0,94995	0,0071	0,9556
Ag-25,000-T	0,009	0,97246	0,009	0,9549
Ag-25,000-P	0,0205	0,92134	0,011	0,85038
Ag-750,000-H	0,0066	0,98456	0,009	0,94232
Ag-750,000-T	0,0184	0,96252	0,0134	0,92531
Ag-750,000-P	0,0267	0,98517	0,0113	0,88634
Ag-2000-H-C	0,0213	0,90459	0,0119	0,98077
Ag-2000-T-C	0,0089	0,96961	0,0102	0,99122
Ag-2000-P-C	0,0123	0,98676	0,0117	0,98096
Ag-5000-H-C	0,0271	0,95951	0,025	0,89056
Ag-5000-T-C	0,0169	0,96965	0,035	0,9261
Ag-5000-P-C	0,0063	0,92089	0,0045	0,75507
Ag-25,000-H-C	0,0217	0,99606	0,0342	0,98993
Ag-25,000-T-C	0,0178	0,96688	0,0235	0,99171
Ag-25,000-P-C	0,024	0,99111	0,0241	0,9775
Ag-750,000-H-C	0,0294	0,96863	0,0131	0,90123
Ag-750,000-T-C	0,0149	0,92474	0,0119	0,9549
Ag-750,000-P-C	0,0181	0,97328	0,0199	0,9661

## 2 Experimental Design, Materials and Methods

### 2.1 Materials

Hepes sodium salt and sodium borohydride were purchased from Acros Organics; 4-Nitroaniline from Merck; Trizma base and Trizma Hydrochloride from Research Organics; hyperbranched poly(ethylene imines); M_w_ = 2000, 5000, 25,000 and 750,000 from BASF under the tradenames Lupasol PR8515, Lupasol G100, Lupasol WF and Lupasol P, M_w_ = 750,000, respectively. Hepes, Tetraethyl Orthosilicate, Silver Nitrate, and 4-Nitrophenol were supplied from Sigma-Aldrich; Disodium Hydrogen Phosphate (Na_2_HPO_4_∙2H_2_O, 99%) from Fluka, and Sodium Dihydrogen Phosphate (NaH_2_PO_4_, 99%) from Riedel de Haën. All compounds did not undergo further purification before use.

### 2.2 Instrumentation

Scanning Electron Microscopy (SEM) micrographs were obtained with the aid of a FEI Inspect microscope with W (Tungsten) filament. UV-Visible spectroscopy for the calculation of the catalytic reduction constants was carried out on a Cary 100 UV–visible spectrophotometer. Thermogravimetric analysis experiments (TGA) under nitrogen flow were performed on a Mettler Toledo TGA/DSC 1 System)(heating rate: 10 °C/min).

### 2.3 Reduction of Silver Cations to Ag Nanoparticles

To 100 ml solutions of PEIs 0.1 mM (approximately 40 mM in primary and secondary amines, 25 ml of AgNO_3_ 0.1 M were added under stirring. The samples remained colorless for the first hour of the experiment at the end of which a slight change to light orange was observed, indicating the beginning of the formation of Ag nanoparticles. The samples were kept under stirring for 8 days, a period followed by a gradient change regarding their color from colorless to dark brown. This procedure was applied for the four different types of PEI with molecular weights of 2000, 5000, 25,000, and 750,000.

### 2.4 Synthesis of SiO_2_-PEI-Ag Nanocatalysts

The second reaction involved the formation of SiO_2_ based on the method proposed by Knecht and Wright [2] modified by our group [3]. 100 ml of each Ag-PEI solution acquired from the first step, was brought to pH 7,5 employing phosphates, Trizma or Hepes, and the conjugate hydrochloride and sodium salts of the latter, respectively. Silver salts, of the pH regulators, when precipitated by non-reduced Ag^+^, were removed by centrifugation. Then, 10 ml of 1 M silicic acid, prepared from the hydrolysis of tetraethyl orthosilicate in 5 mM HCl, were added. Brown precipitates were immediately observed. The samples were centrifuged (10 min 12,000 x g), washed twice with water, and the supernatant was decanted. The final step involved a mild drying of the samples under vacuum over P_2_O_5_. Silica silver nanocatalysts (i.e., without the organic matrice) were obtained by calcination for 3 h at 700 °C under nitrogen. Furthermore, silica-hyperbranched poly(ethylene imine) composites (i.e., without silver nanoparticles) were produced in 20 mM phosphate, trizma, and hepes buffers. Table 1 **c**ontains the classification and nomenclature of all the synthesized materials.

### 2.5 Catalytic Performance Tests

The catalytic properties of the SiO_2_-PEI-Ag nanocatalysts and their calcinated counterparts were assessed by the aid of two standard nitroaromatic compound reduction reactions. The conversion of 4-nitrophenol to 4-aminophenol and 4-nitroaniline to p-phenylenediamine [4]. 5 mg of each sample were dispersed to 50 ml of a 8 ppm aqueous solution of each nitro derivative and then an excess of NaBH_4_ (generally 10 mg) was added. The reaction was monitored at room temperature under continuous stirring by UV-Visible spectroscopy.

## Ethics Statement

The work does not involve human subjects, animal experiments, or data collected from social media platforms.

## CRediT authorship contribution statement

**Michael Arkas:** Conceptualization, Methodology, Formal analysis, Validation, Visualization, Supervision, Project administration. **Marilina Douloudi:** Investigation, Writing – review & editing, Visualization. **Eleni Nikoli:** Writing – review & editing, Visualization. **Georgia Karountzou:** Investigation. **Ioanna Kitsou:** Investigation. **Eleni Kavetsou:** Investigation. **Dimitrios Korres:** Investigation. **Stamatina Vouyiouka:** Methodology, Supervision. **Athena Tsetsekou:** Methodology, Supervision. **Konstantinos Giannakopoulos:** Investigation. **Michaela Papageorgiou:** Investigation, Writing – original draft, Visualization, Funding acquisition.

## Declaration of Competing Interest

The authors declare that they have no known competing financial interests or personal relationships which have or could be perceived to have influenced the work reported in this article.

## Data Availability

TGA-Data (Original data) (Mendeley Data).

## References

[1] Arkas M., Douloudi M., Nikoli E., Karountzou G., Kitsou I., Kavetsou E., Korres D., Vouyiouka S., Tsetsekou A., Giannakopoulos K., Papageorgiou M. (2022). Investigation of two bioinspired reaction mechanisms for the optimization of nano catalysts generated from hyperbranched polymer matrices. React. Funct. Polym..

[2] Knecht M.R., Wright D.W. (2004). Amine-terminated dendrimers as biomimetic templates for silica nanosphere formation. Langmuir.

[3] Arkas M., Kithreoti G., Boukos N., Kitsou I., Petrakli F., Panagiotaki K. (2018). Two completely different biomimetic reactions mediated by the same matrix producing inorganic/organic/inorganic hybrid nanoparticles. Nano Struct. Nano Objects.

[4] Strachan J., Barnett C., Masters A.F., Maschmeyer T. (2020). 4-nitrophenol reduction: probing the putative mechanism of the model reaction. ACS Catal..

[5] Arkas Michael (2022). TGA-Data. Mendelay Data.

